# Do Geographic Barriers and Community Vulnerability Impact PrEP Use Among Transgender Latinas in the US South? Findings from the *ChiCAS* Study

**DOI:** 10.1007/s10461-026-05246-8

**Published:** 2026-07-19

**Authors:** Amanda E. Tanner, Thomas P. McCoy, Lilli Mann-Jackson, Jorge Alonzo, Tamar Goldenberg, Lucero Refugio Aviles, Sandy K. Aguilar-Palma, Eunyoung Y. Song, Amanda Y. Kong, Scott D. Rhodes

**Affiliations:** 1 Department of Public Health Education, University of North Carolina Greensboro, Greensboro, NC USA; 2 Capstone College of Nursing, The University of Alabama, Tuscaloosa, AL USA; 3 Department of Social Sciences and Health Policy, Division of Public Health Sciences, Wake Forest University School of Medicine, Winston-Salem, NC USA; 4 Health Quality Partners, Doylestown Health, Doylestown, PA USA

**Keywords:** Transgender women, Latina, HIV, PrEP

## Abstract

Transgender (trans) Latinas in the United States face disproportionately higher HIV rates and persistently low use of pre-exposure prophylaxis (PrEP) despite awareness. While geographic access to PrEP has been identified as a barrier in other populations, little is known about how spatial access and community-level vulnerability shape PrEP use among trans Latinas in the US South. Using data from the randomized intervention-waitlist control trial of *ChiCAS* (*N* = 144), we examined (1) proximity to Spanish-language PrEP providers, (2) neighborhood vulnerability using the Social Vulnerability Index (SVI), Structural Racism Effect Index (SREI), and the Index of Concentration at the Extremes (ICE), and (3) their associations with PrEP use among trans Latinas at baseline and 6-month follow-up. Multivariable models tested whether baseline contextual factors modified intervention effects. Overall, 15.3% of participants lived in a PrEP desert (≥ 30-minute drive to a Spanish-language PrEP provider). Participants resided in highly vulnerable neighborhoods. Neither PrEP desert status nor community-level vulnerability indices were significantly associated with PrEP use at baseline or follow-up. However, SREI significantly moderated intervention effects (interaction adjusted odds ratio [aOR] = 4.55; 95% confidence interval [CI]:1.08–19.2), with greater intervention impact in areas of higher structural vulnerability. Findings suggest that structural racism, not geographic proximity, more strongly shape PrEP access and use among trans Latinas. Culturally responsive, bilingual interventions like *ChiCAS* may be particularly effective in structurally disadvantaged settings. Future efforts must address structural and social determinants alongside spatial access to achieve PrEP equity.

## Resumen

Las latinas transgénero (trans) en los Estados Unidos experimentan tasas desproporcionadamente altas de VIH y un uso persistentemente bajo de la profilaxis preexposición (PrEP), a pesar de tener conocimiento sobre ella. Se sabe poco acerca de cómo el acceso espacial y la vulnerabilidad a nivel comunitario influyen en el uso de la PrEP entre las latinas trans en el sur de Estados Unidos. Utilizando datos del ensayo *ChiCAS* (*N* = 144), examinamos (1) la proximidad a proveedores de PrEP en español, (2) la vulnerabilidad del vecindario mediante el Índice de Vulnerabilidad Social (SVI), el Índice de Efecto del Racismo Estructural (SREI) y el Índice de Concentración en los Extremos (ICE), y (3) sus asociaciones con el uso de la PrEP entre latinas trans al inicio del estudio y en el seguimiento de 6 meses. En general, el 15.3% de las participantes vivían en un desierto de PrEP (≥ 30 minutos en automóvil hasta un proveedor de PrEP en español). Las participantes residían en vecindarios altamente vulnerables. Ni el estatus de desierto de PrEP ni los índices de vulnerabilidad a nivel comunitario se asociaron con el uso de la PrEP. Estos resultados sugieren que intervenciones como *ChiCAS* pueden ser efectivas en contextos con mayores desventajas estructurales.

## Introduction

In the United States, transgender (trans) women experience a disproportionately high burden of HIV. Current estimates suggest that 14% to 42% of trans women are living with HIV, with racially and ethnically minoritized trans women carrying the heaviest burden [1–4]. A recent cohort study in the Eastern and Southern US estimated HIV incidence at 5.5 per 1,000 person-years, with even higher rates among trans women of color [1]. Trans Latinas are particularly affected, accounting for 35% of the new HIV infections in trans women [4]. Given this burden for trans Latinas, ensuring equitable access to biobehavioral HIV prevention strategies, particularly pre-exposure prophylaxis (PrEP), is critical. PrEP, available in daily oral and long-acting injectable formulations, is a proven, efficacious biomedical prevention tool [5]. While PrEP awareness among trans Latinas is relatively high, use remains low [6–8]. Multilevel barriers affect PrEP use among trans Latinas.

At the individual level, stigma and discrimination based on gender identity, sexual behavior, and perceived immigration status undermine engagement in HIV prevention services [7]. At the spatial and community levels, geographic inaccessibility, often conceptualized as living in a “PrEP desert,” further constrains use. PrEP deserts, commonly defined as residing ≥ 30 minutes one-way travel time from a PrEP provider, are linked to lower use among men who have sex with men (MSM) [9–12]. PrEP deserts are concentrated in the US South and rural areas [9], and trans Latinas in the South and in rural areas likely face similar geographic barriers to PrEP, compounding other structural inequities.

Beyond proximity to PrEP services, community-level vulnerability, shaped by neighborhood composition, poverty, health insurance coverage, and structural racism, drives healthcare resource deprivation [13–17] and likely influences PrEP access. Indices such as the Social Vulnerability Index (SVI), Structural Racism Effect Index (SREI), and Index of Concentration at the Extremes (ICE) quantify these contextual factors and their associations with health outcomes [18–21]. Prior studies have shown that areas with higher vulnerability, including greater proportions of residents living in poverty, lacking insurance, or identifying as Latine or Black, have fewer PrEP providers [9, 11]. Community-level vulnerability affects healthcare access [13, 17] and thus, could have implications for PrEP use among trans Latinas.

### Study Purpose and Research Questions (RQs)

Using data from the *ChiCAS: Chicas Creando Acceso a la Salud* (*Girls Creating Access to Health*) randomized intervention-waitlist control trial [8], we explored how geospatial access and community-level vulnerability influence PrEP use among trans Latinas in the US South. Specifically, we address the following research questions:

### PrEP access, community vulnerability, and assocations with PrEP use

RQ1: What proportion of participants live in a PrEP desert at baseline?

RQ2: What is the distribution of community-level vulnerability in the neighborhoods where trans Latinas in this sample live?

RQ3: How does proximity to Spanish-speaking PrEP providers and neighborhood vulnerability relate to PrEP use at baseline and follow-up of *ChiCAS* intervention?

RQ4: Do Spanish-language PrEP access and community-level vulnerability at baseline moderate the effects of the *ChiCAS* intervention on PrEP use at follow-up?

## Methods

### Study Design

This analysis draws on baseline and 6-month follow-up data from a randomized intervention-waitlist control trial designed to evaluate the efficacy of the *ChiCAS* intervention. *ChiCAS* is a small-group, two-session intervention, delivered in person or virtually, to promote PrEP use, consistent condom use, and medically supervised gender-affirming hormone therapy among Spanish-speaking, HIV-seronegative trans Latinas who have sex with men [22–24]. The intervention demonstrated efficacy in improving PrEP use in this population [8].

Between July 2019 and July 2021, trans Latinas were recruited across North and South Carolina using a multi-pronged outreach strategy. Recruitment included dissemination of study information and in-person outreach at nightclubs, LGBTQ+ organizations, community colleges, and Latine-owned businesses, as well as social media advertising and word-of-mouth referrals [8, 24]. Eligibility criteria included: (1) identifying as a trans woman or having been assigned male sex at birth and self-identifying as female, (2) identifying as Hispanic/Latina, (3) being 18 years of age or older, (4) reporting sex with at least one man in the past 6 months, (5) being HIV negative (self-report verified by rapid testing; before COVID-19 this was done via blood test administered by phlebotomist (rapid INSTI HIV Test by bioLytical) and after COVID-19 onset this was done via oral, self-administered test (OraQuick In-Home HIV Test) [25]), (6) speaking fluent Spanish, and (7) providing informed consent. Individuals who had participated in any HIV prevention interventions in the past 12 months were ineligible. We enrolled 144 trans Latinas after screening at total of 162 people; one did not meet inclusion criteria, 11 were not available/schedule conflict, and 5 were not able to participate given COVID-19 stay-at-home order. Notably, mail-order PrEP became available over the course of the study, but no participants reported use. Participants completed interviewer-administered REDCap assessments at baseline and 6-month follow-up and were compensated $30 and $40, respectively, for their time. The Institutional Review Board of Wake Forest University School of Medicine provided human subject oversight (IRB00040441).

### Measures

#### Sociodemographic and Behavioral Characteristics

We assessed sociodemographic characteristics at baseline, including age, sexual orientation, gender identity, language, time in the United States, country of origin, educational attainment, employment, and income using measures that have been used with Spanish-speaking trans Latinas [25, 26]. Age was measured as a continuous variable in years. Standardized sexual orientation and gender identity (SOGI) questions were used to assess sexual orientation and gender [27]. Spanish and English fluency were assessed using the item, “In which language would you consider yourself to be fluent?” Time in the United States was measured as a continuous variable in years. Educational attainment was assessed and responses were categorized into less than high school diploma or General Educational Development (GED) equivalent, and receipt of a high school diploma or GED or more. Employment was assessed with responses categorized into less than year-round employment, and year-round employment. Participants indicated their monthly income from all sources. Participants reported behaviors. They were asked about condom use in past 3 months, separately by behavior (anal and/or vaginal sex) and for male or female partners (for both insertive and receptive sex); answer profiles were then dichotomized (always/consistent condom use across activity or sexually inactive vs. inconsistent use). Finally, participants were asked about history of STI diagnosis (gonorrhea, chlamydia, and syphilis) and needle sharing in the past 12 months.

#### PrEP Use and Living in a PrEP Desert

Current PrEP use was assessed by asking participants whether they were currently using PrEP at both baseline and 6-month follow-up (yes/no). To determine whether participants lived in a PrEP desert, we geocoded participants’ baseline residential addresses along with the addresses of all PrEP providers in North Carolina and the Upstate and Midlands regions of South Carolina, where participants resided. Provider data were abstracted from the PrEP Locator (https://preplocator.org/), a national directory that includes public and private PrEP providers from the same year as data collection. Each location was further coded for Spanish-language service availability, creating a list of Spanish-language PrEP providers. Using the ArcGIS Desktop Roads and Highways extension, we calculated driving distance and travel time from each participant’s residence to the nearest provider offering Spanish-language services (Environmental Systems Research Institute [ESRI], Release 10.1, 2012, Redlands, CA). Consistent with prior geospatial PrEP access research [10–12], participants residing ≥ 30 min one-way travel time from the closest Spanish-language PrEP provider were classified as living in a PrEP desert.

#### Community-Level Vulnerability

We assessed community-level vulnerability at baseline by census tract using three indices. First, we downloaded the SVI, a place-based composite index developed by the Centers for Disease Control and Prevention (CDC) that uses 16 US Census variables from the American Community Survey (ACS) grouped into four themes—socioeconomic status, household characteristics, racial/ethnic minority status, and housing/transportation—to produce an overall score ranging from 0 to 1, with higher values indicating greater vulnerability [18]. We used the SVI data that was closest in time to the timing of the baseline data collection. However, because of changes in Federal Information Processing Standards (FIPS) codes, SVI scores could not be assigned for 28% of participants. These changes included census tracts or counties being split into multiple units (each with a new FIPS code), combined into larger units, or retired altogether because of boundary adjustments.

Second, we used the SREI which incorporates nine census tract-level domains to capture levels of structural racism and economic inequality. Scores are standardized with a mean of 0 and standard deviation of 1; positive scores indicate areas with fewer resources and negative scores reflect areas with greater access to resources [19].

Finally, we used the ICE. ICE quantifies the degree to which populations are spatially segregated or concentrated at the most privileged or most deprived ends of the racial-socioeconomic spectrum. ICE includes three indices: race, income, and combined race and income. Values range from − 1 (100% of population in the most deprived group) to + 1 (100% in the most privileged group) [20, 21]. This analysis used the combined race–income ICE, with higher values representing a greater concentration of high-income Non-Hispanic/Latine White residents relative to low-income non-White residents within census track, as defined by ACS household income categories closest to the 20th (for low) and 80th (for high) U.S. household income percentiles [21].

### Data Analysis

Descriptive statistics including mean (*M*), standard deviation (*SD*), frequency (*n*), and percent (%) were computed to summarize study measures prior to inferential analysis. For PrEP desert status and PrEP use prevalence, point estimates and corresponding 95% Wilson confidence intervals (CIs) were calculated [28]. Spearman correlation coefficients (*r*ₛ) were used to assess pairwise associations among community-level vulnerability indices (SVI, SREI, and combined race-income ICE). For bivariate associations with PrEP use, Chi-square or Fisher’s exact tests were used for categorical variables (e.g., PrEP desert status), and *t*-tests or Wilcoxon rank sum tests were used for continuous variables (e.g., community-level vulnerability indices).

For modeling PrEP use at baseline and at 6 month follow-up, age, less than high school education vs. higher, employed year-round vs. otherwise, Mexico country of origin, virtual vs. in-person intervention participation, wave of intervention, and intervention vs. delayed intervention (group) were adjusted for (as done in [8]) and was expanded to additionally include PrEP desert status or community-level vulnerability index along with the pairwise interactions of intervention group × desert status or intervention group × vulnerability index. For community-level vulnerability indices, these were first centered on their sample means before creating pairwise interactions to mitigate potential multicollinearity [18–21, 29] and was checked with VIFs and eigen analysis [30]. Multicollinearity checks showed no evidence of substantial collinearity (e.g., range VIFs 1.0–2.9 across modeling). Separate logistic models examined associations between PrEP use with each index and PrEP desert status. Interactions were evaluated and because they were not statistically significant, subsequent final models included only uncentered main effects. Sensitivity analyses for missing data were performed using multiple imputation via fully conditional specification with 200 imputations, and pooled parameter estimates and test statistics (*t* versus complete-case *F* in SAS PROC MIANALYZE) presented. Analyses were performed using ArcGIS Desktop v10.7.1 and SAS v9.4 (SAS Institute, Cary, NC). A two-sided *p*-value < 0.05 was considered statistically significant.

## Results

### Participant Sociodemographics, Behavioral Characteristics, and PrEP Use

The mean age of the study participants was 33.1 years (*SD* = 9.4). Participants self-identified as transgender women (97%), or female (2%). Sexual orientation was reported as heterosexual (81%), bisexual (10%), gay (6%), and “something else” (3%). Approximately half had less than a high school education or GED equivalent; 65% were employed year-round; and 80% reported earning less than $2,000 per month. Most participants were foreign born (88%). Foreign-born participants had been living in the United States for a mean of 15.4 years (*SD* = 8.7). 70% reported speaking only or mostly Spanish. Close to two-thirds (64%) reported inconsistent condom use, 16% had ever had a STI, and one participant reported sharing needles in the last 12 months (see Table 1).

**Table 1:** Sociodemographic and behavioral characteristics of *ChiCAS* participants* (*N* = 144)

Sociodemographic characteristic^1^	*n* (%) or Mean ± SD
Age in years	33.1 ± 9.4
Years living in US among foreign born (*n* = 124)	15.4 ± 8.7
Country of origin	
Mexico	92 (63.9)
United States	17 (11.8)
Other	35 (24.3)
Education (*n* = 143)	
Less than high school education or GED equivalent	71 (49.7)
High school or GED equivalent or above	72 (50.4)
Employment (*n* = 143)	
Employed year round	93 (65.0)
Other	50 (35.0)
Monthly income (*n* = 141)	
None	30 (21.3)
<$1,000	26 (18.4)
$1,000-$1,999	57 (40.4)
≥$2,000	28 (19.9)
Condom use last 3 months (*n* = 135)	
Inconsistent use	86 (63.7)
Consistent/always/inactive	49 (36.3)
Ever had any STIs (*n* = 138)	22 (15.9)
Needle sharing, past 12 mos. (*n* = 143)	1 (0.7)
Community-level vulnerability	
SVI (*n* = 103)	0.82 ± 0.16
SREI (*n* = 136)	0.95 ± 0.78
ICE (*n* = 137)	0.05 ± 0.06
Living in a Spanish-speaking PrEP desert (> 30 min)	22 (15.3)
Pre-exposure prophylaxis (PrEP) use	
At baseline (*n* = 141)	12 (8.5)
At 6-month follow-up (*n* = 136)	31 (22.8)

^1^*n* for each characteristic may not equal 144 due to missing data; % are out of available *n* for characteristic; all characteristics are from baseline data except for 6-months

Follow-up PrEP use

*SD* standard deviation, *GED* general educational development

 RQ1: What proportion of participants live in a PrEP desert at baseline?

**Fig. 1:**
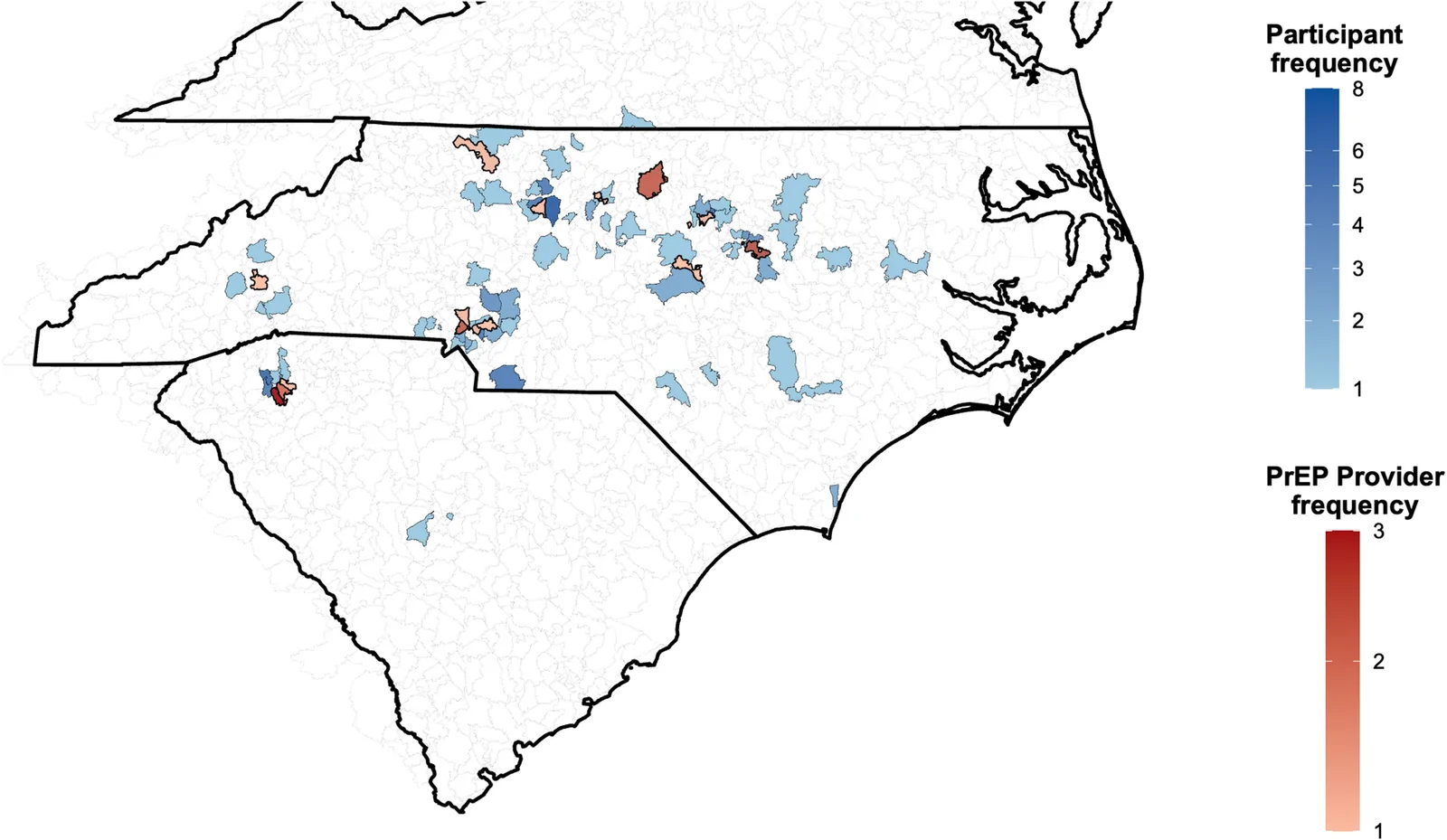
Map of PrEP provider locations with Spanish-language services and study participant residence by ZCTA at baseline. *ZCTA: United States Census Bureau ZIP Code Tabulation Area

Overall, 15.3% (*n* = 22; 95% CI=[10.3%, 22.0%]) of the participants were living in a PrEP desert at baseline. Figure 1 illustrates the geographic distribution of Spanish-language PrEP providers relative to participant residences across North and South Carolina using United States Census Bureau ZIP Code Tabulation Area (ZCTA) to protect participant identity. Most urban areas within the study catchment, including Asheville, Greenville-Spartanburg, Charlotte-Mecklenburg, the Triad (Winston-Salem, Greensboro, High Point), Burlington, and the Triangle (Raleigh, Chapel Hill, Durham), had at least one Spanish-language PrEP provider. In contrast, Columbia, South Carolina, had no identified Spanish-speaking PrEP providers.

RQ2: What are the patterns of community-level vulnerability in the neighborhoods where trans Latinas in this sample live?

Data on community-level vulnerability were available for most participants. SVI values were available for 103 (71.5%), with a mean = 0.82 (*SD* = 0.16), indicating that participants primarily lived in highly socially vulnerable areas. SREI values were available for 136 participants (94.4%), with a mean = 0.95 (*SD* = 0.78), suggesting that, on average, participants resided in neighborhoods with fewer structural resources, though there was substantial variability across census tracts. ICE values were available for 137 participants (95.1%), with a mean of 0.05 (*SD* = 0.06), suggesting relatively mixed neighborhood composition with limited racialized economic polarization. The three indices were moderately to strongly and significantly correlated, indicating that they capture related but distinct dimensions of community vulnerability: SVI and SREI (*r*_s_=0.68, *t* = 9.29, *p* < 0.001), SVI and ICE (*r*_s_=-0.55, *t*=-6.46, *p* < 0.001), and SREI and ICE (*r*_s_=-0.59, *t*=-8.47, *p* < 0.001).

RQ3: How does proximity to Spanish-speaking PrEP providers and neighborhood vulnerability relate to PrEP use at baseline and follow-up?

No statistically significant associations were observed between proximity to Spanish-language PrEP providers and PrEP use at either baseline or 6-month follow-up. At baseline, 1 of 12 (8.3%) participants using PrEP lived in a PrEP desert compared with 21 of 105 (20.0%) participants not using PrEP (OR = 0.44, 95% CI for OR=[0.05, 3.57], Fisher’s Exact *p* = 0.69). Similarly, living in a PrEP desert was not associated with PrEP use at 6-month follow-up (OR = 0.75, 95% CI for OR=[0.23, 2.43], *p* = 0.78). None of the community-level vulnerability measures were associated with baseline PrEP use (SVI *z* = 0.17, *p* = 0.87; SREI *z*=-0.63, *p* = 0.53; ICE_ri_ *z*=-1.31, *p* = 0.19). Additionally, the SVI was not significantly associated with PrEP use at follow-up (*z*=-0.64, *p* = 0.52); similarly, there were non-significant associations between each of the four sub domains and use at follow-up (range *z*=-0.81 to 1.34, *p* = 0.18 to 0.42). The SREI was also not significantly associated with PrEP use at follow-up (*z* = 1.20, *p* = 0.23). Finally, ICE values were approaching significance but not significantly associated with PrEP use at follow-up (*z*=-1.80, *p* = 0.07). These findings were also consistent with multivariable logistic main effects modeling of PrEP use at baseline and follow-up (see Tables 2 and 3).

**Table 2:** Logistic regression modeling for baseline PrEP use

Effect	Model 1 aOR (95% CI)	Model 2 aOR (95% CI)	Model 3 aOR (95% CI)	Model 4 aOR (95% CI)
Age at baseline (years)	1.02 (0.95, 1.10)	1.01 (0.94, 1.09)	1.02 (0.94,1.10)	1.01 (0.94, 1.09)
< High school education	0.10* (0.01, 0.71)	0.12* (0.02, 0.79)	0.12* (0.02, 0.78)	0.10* (0.01, 0.74)
Employed year-round	1.13 (0.26, 4.93)	0.93 (0.22, 3.97)	0.84 (0.20, 3.47)	0.89 (0.21, 3.77)
Born in Mexico	0.57 (0.13, 2.39)	0.60 (0.14, 2.50)	0.58 (0.14, 2.40)	0.56 (0.13, 2.38)
Participated virtually	0.42 (0.09, 2.02)	0.44 (0.09, 2.20)	0.43 (0.09, 2.09)	0.50 (0.10, 2.53)
Intervention vs. delayed intervention	1.24 (0.33, 4.64)	1.50 (0.41, 5.49)	1.43 (0.39, 5.20)	1.25 (0.34, 4.63)
Model 1: PrEP desert (yes vs. no)	0.26 (0.03, 2.64)	–	–	–
Model 2: SVI	–	4.32 (0.04, 441.7)	–	–
Model 3: SREI	–	–	0.82 (0.34, 1.98)	–
Model 4: ICEri	–	–	–	< 0.01 (< 0.01, 21.3)

Numbers are adjusted odds ratio (aOR), (95% Confidence Intervals (CI))

*p < 0.05, **p < 0.01, ***p < 0.001

*PrEP* pre-exposure prophylaxis, *SVI* social vulnerability index, *SREI* structural racism effect index, *ICEri* index of concentration at the extremes (race × income)

Results are after multiple imputation for missing data with 200 imputations

**Table 3:** Logistic regression modeling for 6-months PrEP use

Effect	Model 1	Model 2	Model 3	Model 4
Age at baseline (years)	0.99 (0.94, 1.05)	0.99 (0.93, 1.05)	0.98 (0.93, 1.04)	0.99 (0.94, 1.05)
< High school education	2.43 (0.84, 7.07)	2.26 (0.79, 6.46)	2.46 (0.85, 7.14)	2.15 (0.76, 6.11)
Employed year-round	0.65 (0.22, 1.89)	0.82 (0.29, 2.37)	0.83 (0.29, 2.40)	0.80 (0.28, 2.29)
Born in Mexico	0.98 (0.31, 3.09)	0.92 (0.29, 2.89)	0.86 (0.27, 2.74)	0.85 (0.27, 2.69)
Participated virtually	0.84 (0.27, 2.64)	0.93 (0.30, 2.87)	0.95 (0.30, 2.97)	0.95 (0.30, 2.97)
Intervention vs. delayed intervention	4.01** (1.44, 11.2)	4.08** (1.46, 11.4)	4.40** (1.54, 12.6)	3.84** (1.40, 10.5)
Model 1: PrEP desert (yes vs. no)	1.75 (0.40, 7.64)	–	–	–
Model 2: SVI	–	6.93 (0.23, 206.3)	–	–
Model 3: SREI	–	–	1.93 (0.98, 3.81)	
Model 4: ICEri	–	–	–	0.01 (< 0.01, 36.6)

Numbers are adjusted odds ratio (aOR), (95% Confidence Intervals (CI))

*p < 0.05, **p < 0.01, ***p < 0.001

*PrEP* pre-exposure prophylaxis, *SVI* social vulnerability index, *SREI* structural racism effect index, *ICEri* index of concentration at the extremes (race × income)

Results are after multiple imputation for missing data with 200 imputations and additionally adjusted for PrEP use at baseline

RQ4: Do Spanish-language PrEP access and community-level vulnerability at baseline moderate the effects of the *ChiCAS* intervention on PrEP use at follow-up?

Modification of intervention effects for PrEP use was not significant for: PrEP desert status (*t*=-1.17, *p* = 0.24), SVI (*t* = 0.27, *p* = 0.79), ICE (*t*=-0.44, *p* = 0.66), or SREI (*t*=-1.70, *p* = 0.09) in pooled logistic modeling results after multiple imputation for missing data. Although complete-case analysis with listwise deletion suggested a statistically significant effect modification by SREI for PrEP use at follow-up (*F* = 4.35, *p* = 0.04), because this finding was sensitive to how observed missing data were treated, there is uncertainty around this moderating effect and future research could examine for further determination.

## Discussion

Most trans Latinas in this study lived within relatively close geographic proximity to a Spanish-language PrEP provider, suggesting that physical proximity to PrEP services may be less of a barrier than often assumed for trans Latinas in the US South. However, despite relatively high geographic access, PrEP use remained modest, with 8.5% of participants reporting current use at baseline and 22.8% at follow-up. While this increase represents an important improvement, many trans Latinas who could benefit from PrEP were not using it, highlighting the need to better understand and address barriers beyond geographic access. Measures of community-level vulnerability [18–21] revealed that participants predominantly lived in communities marked by high social vulnerability, economic segregation, and structural racism, which likely create barriers beyond spatial proximity, including those linked to discrimination, stigma, and immigration concerns.

Across analyses, however, neither geographic proximity to Spanish-language PrEP services nor indices of neighborhood vulnerability (i.e., SVI, SREI, and ICE) were independently associated with PrEP use at baseline or follow-up. Although some areas within the study catchment lacked Spanish-language PrEP services, only 15.3% of participants resided in PrEP deserts, defined as residing ≥ 30 minutes one-way travel time from a Spanish-language PrEP provider [10, 11], suggesting that geographic travel time may not be the primary barrier to PrEP use among trans Latinas in this area. Other structural and social determinants, such as discrimination, immigration-related fears, and stigma may play more critical roles in shaping PrEP use [13–17]. These findings further underscore the complexity of PrEP use, suggesting that contextual inequities, while pervasive, may influence use indirectly, through cumulative structural barriers and intersectional forms of marginalization, rather than via proximity to PrEP access alone.

Together, these findings suggest that improving spatial access alone is unlikely to close gaps in PrEP use among trans Latinas. Instead, interventions must be multilevel and equity-focused, addressing social, structural, and systemic barriers that constrain HIV prevention access. The *ChiCAS* intervention appears especially promising for reaching those in structurally marginalized environments, where its culturally and linguistically responsive design may counteract the effects of resource deprivation and structural racism. Future research should further explore how spatial, social, structural, and systemic factors interact with individual-level determinants to shape PrEP trajectories, and test interventions that integrate community engagement, policy advocacy, and structural change to achieve sustained and equitable HIV prevention outcomes.

### Study Strengths and Limitations

This analysis offers novel insights into how spatial access and community-level vulnerability influence PrEP use and intervention effectiveness among trans Latinas in the US South, an underserved population disproportionately affected by HIV. By integrating geospatial analyses, structural indices, and intervention data from a randomized trial, the study advances understanding of how structural racism and contextual inequities shape HIV prevention outcomes. Several limitations should be noted. First, the study sample was limited to Spanish-speaking trans Latinas residing in North and South Carolina, which may restrict generalizability to trans Latinas in other geographic regions or those who primarily speak English. Second, cross-sectional measures of community-level vulnerability and spatial access were based on baseline residential addresses, which may not capture mobility or changes in context over time. Third, PrEP use was self-reported, which may be subject to social desirability or recall bias, though this is mitigated by prior research validating self-report in similar populations [31–34]. Fourth, some participants (28%) could not be assigned an SVI score due to changes in FIPS codes, potentially introducing bias or limiting comparisons across indices although sensitivity analyses after multiple imputation yielded similar conclusions for SVI effects. Fifth, we used a binary measure based on a ≥ 30-minute one-way travel time from a PrEP provider to define a PrEP desert [12] and limited this definition to Spanish-speaking PrEP providers. There are other measures of accessibility (e.g., continuous number of minutes or distance to reach a PrEP provider) [10], and there may be other accessibility attributes that were not fully captured with our measures, such as availability to public transportation and or mail-order options to access PrEP services (regardless of living in PrEP desert) and proximity to (a) preferred types of PrEP providers (pharmacies vs. medical facility) and (b) high quality, gender-affirming, and trustworthy care. Finally, statistical power to detect effect sizes may have been limited given the modest sample size and non-significant findings should be interpreted with caution. Despite these limitations, this study is among the first to examine structural racism metrics with PrEP use in a trans Latina population and underscores the need for multilevel, equity-focused interventions that address social, spatial, and structural barriers.

## Conclusions

The high HIV burden among trans Latinas underscores an urgent need for evidence-based HIV prevention strategies, including those that promote biomedical tools such as PrEP [7]. The combination of persistently low PrEP use and residence in disadvantaged neighborhoods points to the need for multilevel, equity-focused approaches that integrate cultural responsiveness, community engagement, and structural competency into HIV prevention programming. Future efforts should prioritize interventions and policies that explicitly address structural vulnerabilities, including economic segregation, racialized resource inequities, and systemic exclusion from care, while sustaining investments in linguistically and culturally responsive services. Continued research is needed to clarify how geographic access [10, 11] and community-level vulnerability interact within the changing geopolitical landscape to influence PrEP use among trans Latinas. By integrating spatial analysis, structural measures, and community-engaged approaches that center the voices and experiences of trans Latinas, future research can advance health equity and ensure that HIV prevention strategies are both effective and responsive to those most affected.
